# Curcumin induces chemo/radio-sensitization in ovarian cancer cells and curcumin nanoparticles inhibit ovarian cancer cell growth

**DOI:** 10.1186/1757-2215-3-11

**Published:** 2010-04-29

**Authors:** Murali M Yallapu, Diane M Maher, Vasudha Sundram, Maria C Bell, Meena Jaggi, Subhash C Chauhan

**Affiliations:** 1 Cancer Biology Research Center, Sanford Research/University of South Dakota, Sioux Falls, SD 57105, USA; 2 Department of Obstetrics and Gynecology, Sanford School of Medicine, University of South Dakota, Sioux Falls, SD 57105, USA

## Abstract

**Background:**

Chemo/radio-resistance is a major obstacle in treating advanced ovarian cancer. The efficacy of current treatments may be improved by increasing the sensitivity of cancer cells to chemo/radiation therapies. Curcumin is a naturally occurring compound with anti-cancer activity in multiple cancers; however, its chemo/radio-sensitizing potential is not well studied in ovarian cancer. Herein, we demonstrate the effectiveness of a curcumin pre-treatment strategy for chemo/radio-sensitizing cisplatin resistant ovarian cancer cells. To improve the efficacy and specificity of curcumin induced chemo/radio sensitization, we developed a curcumin nanoparticle formulation conjugated with a monoclonal antibody specific for cancer cells.

**Methods:**

Cisplatin resistant A2780CP ovarian cancer cells were pre-treated with curcumin followed by exposure to cisplatin or radiation and the effect on cell growth was determined by MTS and colony formation assays. The effect of curcumin pre-treatment on the expression of apoptosis related proteins and β-catenin was determined by Western blotting or Flow Cytometry. A luciferase reporter assay was used to determine the effect of curcumin on β-catenin transcription activity. The poly(lactic acid-*co*-glycolic acid) (PLGA) nanoparticle formulation of curcumin (Nano-CUR) was developed by a modified nano-precipitation method and physico-chemical characterization was performed by transmission electron microscopy and dynamic light scattering methods.

**Results:**

Curcumin pre-treatment considerably reduced the dose of cisplatin and radiation required to inhibit the growth of cisplatin resistant ovarian cancer cells. During the 6 hr pre-treatment, curcumin down regulated the expression of Bcl-X_L _and Mcl-1 pro-survival proteins. Curcumin pre-treatment followed by exposure to low doses of cisplatin increased apoptosis as indicated by annexin V staining and cleavage of caspase 9 and PARP. Additionally, curcumin pre-treatment lowered β-catenin expression and transcriptional activity. Nano-CUR was successfully generated and physico-chemical characterization of Nano-CUR indicated an average particle size of ~70 nm, steady and prolonged release of curcumin, antibody conjugation capability and effective inhibition of ovarian cancer cell growth.

**Conclusion:**

Curcumin pre-treatment enhances chemo/radio-sensitization in A2780CP ovarian cancer cells through multiple molecular mechanisms. Therefore, curcumin pre-treatment may effectively improve ovarian cancer therapeutics. A targeted PLGA nanoparticle formulation of curcumin is feasible and may improve the *in vivo *therapeutic efficacy of curcumin.

## Background

Ovarian cancer is the most lethal gynecological cancer and the fifth most common cause of cancer mortality in women in the United States: in 2009 it is estimated that 21,550 women will be diagnosed with ovarian cancer and 14,600 women will die due to this disease [1]. A high percent of women with ovarian cancer are diagnosed at an advanced stage (67%) and have a 5 year survival rate of only 46% [1]. The usual treatment modality involves surgical cytoreduction followed by treatment with a combination of platinum (cisplatin or carboplatin) and taxane based therapies. This is effective in 60-80% of patients; however, the majority of women with advanced disease will have cancer recurrence [2,3]. Unfortunately, almost all relapsing ovarian cancers eventually develop platinum resistance and patients with resistant tumors have a median survival time of 6 months, with only 27% living longer than 12 months [4]. In addition to improving diagnosis of ovarian cancer, there is an urgent need to develop effective therapeutic modalities for advanced stage drug resistant ovarian cancer.

Although the mechanism of resistance to cisplatin has been widely studied *in vitro*, the actual reasons underlying cisplatin resistance *in vivo *is still not well understood. Cisplatin functions primarily by forming DNA adducts that inhibit cell replication and induce apoptosis if the DNA damage is not repaired by the cell. Recently, it has been suggested that while initial sensitivity to cisplatin is *via *nonfunctional DNA repair genes (i.e. BRCA1/2), cisplatin resistance may be acquired through a gain of function in BRCA1/2 [5]. Independent of the mechanism of resistance, inhibition of cell death *via *apoptosis is an important event leading to cisplatin resistance. Another important aspect limiting the use of cisplatin is the negative side effects which accumulate in severity with multiple cisplatin treatments and include gastrointestinal distress, kidney and nerve damage, hearing loss, and bone marrow suppression [2,3,6]. Additionally, treatment of ovarian cancer with radiation is limited due to gastrointestinal toxicity [6]. While significant progress has been made in developing targeted radioimmunotherapy (RIT), current drawbacks to this therapy include toxicity and resistance to radiation [7,8].

One strategy to improve the effectiveness and limit the toxicity of cisplatin and/or radiation therapy is to induce chemo/radio-sensitization in cancer cells. A number of natural dietary phytochemicals, such as curcumin, quercetin, xanthorrhizol, ginger, green tea, genistein, etc., are candidates for inducing chemo/radio-sensitization of cancer cells [9-11]. Among these agents, curcumin (diferuloyl methane), a polyphenol derived from the rhizomes of tumeric, *Curcuma longa*, has received considerable attention due to its beneficial chemopreventive and chemotherapeutic activity *via *influencing multiple signaling pathways, including those involved in survival, growth, metastasis and angiogenesis in various cancers [12-15]. Importantly, curcumin has demonstrated no toxicity to healthy organs at doses as high as 8 grams/day [16]. However, the low bioavailability and poor pharmacokinetics of curcumin limits its effectiveness *in vivo *[17]; therefore, we have developed a PLGA nanoparticle formulation of curcumin (Nano-CUR) to provide increased bioavailability as well as antibody conjugation abilities for targeted delivery of curcumin into tumors.

Given the need for therapies to treat cisplatin resistant ovarian cancer, we investigated the effect of curcumin pre-treatment on a cisplatin resistant ovarian cancer cell line model. We demonstrate, for the first time, that curcumin pre-treatment sensitizes A2780CP cells (which are cisplatin resistant) to cisplatin and radiation treatment. Curcumin pre-treatment dramatically inhibits proliferation and clonogenic potential of cisplatin resistant cells in the presence of low levels of cisplatin or radiation. We also identified molecular pathways involved in curcumin mediated sensitization to cisplatin/radiation induced apoptosis. This study advances the understanding regarding the molecular mechanisms involved in curcumin mediated chemo/radio-sensitization in ovarian cancer cells.

## Materials and methods

### Cell culture and drugs

A2780 and A2780CP (resistant to cisplatin) paired cells [18] were generously provided by Dr. Stephen Howell, University of California, San Diego. These cells were maintained as monolayer cultures in RPMI-1640 medium (HyClone Laboratories, Inc. Logan, UT) supplemented with 10% fetal bovine serum (Atlanta Biologicals, Lawrenceville, GA) and 1% penicillin-streptomycin (Gibco BRL, Grand Island, NY) at 37°C in a humidified atmosphere (5% CO_2_). Curcumin (≥ 95% purity, (E, E)-1,7-bis(4-Hydroxy-3-methoxyphenyl)-1,6-heptadiene-3,5-dione, Sigma, St. Louis, MO) was stored at -20°C as 10 mM stock solution in DMSO and protected from light. Cisplatin (cis-Diammineplatinum(II) dichloride, Sigma) was stored at 4°C as 10 mM stock solution in 0.9% saline.

### Cell growth and viability

Cells were seeded at 5,000 per well in 96-well plates, allowed to attach overnight and different concentrations (2.5-40 μM) of curcumin or cisplatin diluted in medium were added. DMSO and PBS containing medium served as the respective controls. In another set, cells were treated for 6 hrs with 10 or 20 μM curcumin in medium and followed by cisplatin treatment (2.5-40 μM). DMSO-PBS medium was used as a control. The anti-proliferative effect of these drugs was determined at 2 days with a MTS based colorimetric assay (CellTiter 96 AQ_eous _One Solution Cell Proliferation Assay, Promega, Madison, WI). The reagent (20 μL/well) was added to each well and plates were incubated for 2 hrs at 37°C. The color intensity was measured at 492 nm using a microplate reader (BioMate 3 UV-Vis spectrophotometer, Thermo Electron Corporation, Waltham, MA). The anti-proliferative effect of each treatment was calculated as a percentage of cell growth with respect to the appropriate controls after subtracting intensity values for curcumin, DMSO, PBS and DMSO-PBS in medium without cells. Phase contrast microscope cell images were taken on an Olympus BX 41 microscope (Olympus, Center Valley, PA).

### Colony formation assay

For this assay, cells were seeded at 500 cells per 100 mm culture dish and allowed to attach overnight. The cells were treated with curcumin or cisplatin or with a pre-treatment of curcumin followed by cisplatin treatment and maintained under standard cell culture conditions at 37°C and 5% CO_2 _in a humid environment. After 8 days, the dishes were washed twice in PBS, fixed with methanol, stained with hematoxylin (Fisher Scientific, Pittsburgh, PA), washed with water and air dried. The number of colonies was determined by imaging with a Multimage™ Cabinet (Alpha Innotech Corporation, San Leandro, CA) and using AlphaEase Fc software. The percent of colonies was calculated using the number of colonies formed in treatment divided by number of colonies formed in DMSO or PBS or DMSO-PBS control.

### Radiation

Cells were seeded at 200 per well in 6 well plates and allowed to attach overnight. These cells were treated with different concentrations of curcumin for 6 hrs and exposed to 1-5 Gy dose of radiation. A 1060 kV industrial RS-2000 Biological X-ray irradiator (Radiation Source, Alpharetta, GA) was used to irradiate the cultures at room temperature. The machine was operated at 25 mA. The dose rate with a 2 mm Al and 1 mm Be filter was ~1.72 Gy/min at a focus surface distance of 15 cm. Cells treated with different concentrations of curcumin or radiation alone was used as controls. These cells were maintained under standard cell culture conditions at 37°C and 5% CO_2 _in a humid environment. After 8 days, the colonies were counted as described earlier.

### Immunoblot assay

Following treatment, cells were processed for protein extraction and Western blotting using standard procedures as described earlier [19]. Briefly, 800,000 cells per 100 mm cell culture dish were plated, allowed to attach overnight and treated with curcumin or cisplatin or pretreated with curcumin followed by cisplatin. After 48 hrs cells were washed twice with PBS, lysed in SDS buffer (Santa Cruz Biotechnology, Santa Cruz, CA) and kept at 4°C for 30 min. Cell lysates were passed through one freeze-thaw cycle and sonicated on ice for 30 sec (Sonic Dismembrator Model 100, Fisher Scientific) and the protein concentration was normalized using SYPRO Orange (Invitrogen, Carlsbad, CA). The cell lysates were heated at 95°C for 5 min, cooled down to 4°C, centrifuged at 14,000 rpm for 3 min and the supernatants were collected. SDS-PAGE (4-20%) gel electrophoresis was performed and the resolved proteins were transferred onto PVDF membrane. After rinsing in PBS, membranes were blocked in 5% nonfat dry milk in TBS-T (Tris buffered saline containing 0.05% Tween-20) for 1 hr and incubated with Bcl-X_L_, Mcl-1, Caspase 3, 7 and 9, Poly (ADP-ribose) polymerase (PARP), β-catenin, c-Myc and β-actin specific primary antibodies (Cell Signaling, Danvers, MA) overnight at 4°C. The membranes were washed (4 × 10 min) in TBS-T at room temperature and then probed with 1:2000 diluted horseradish peroxidase-conjugated goat anti-mouse or goat anti-rabbit secondary antibody (Promega) for 1 hr at room temperature and washed (5 × 10 min) with TBS-T. The signal was detected with the Lumi-Light detection kit (Roche, Nutley, NJ) and a BioRad Gel Doc (BioRad, Hercules, CA).

### Annexin V staining

Cells were plated, allowed to attach overnight and treated with cisplatin or curcumin alone or pre-treated with curcumin for 6 hrs and followed by cisplatin treatment for an additional 42 hrs. Both adherent and floating cells were collected, washed with PBS, suspended in Annexin V binding buffer, stained with Annexin V-PE (BD Biosciences, San Diego, CA) and analyzed by flow cytometry using an Acuri C6 flow cytometer (Accuri Cytometers, Inc., Ann Arbor, MI).

### TOPFlash reporter assay

The β-catenin-TCF transcription activity was measured using a luminescence reporter assay as described earlier [20]. In short, 200,000 cells were plated per well in a 12 well plate for 16 hrs prior to transient transfection with reporter construct TOPFlash or FOPFlash (Gift from Dr. R. Moon, Washington University) and cotransfected with Renilla luciferase (pRL-TK, Promega). After 3 hrs of transfection, the wells were treated with either 20 μM curcumin, 5 μM cisplatin or a 6 hr pre-treatment with 20 μM curcumin followed by treatment with 5 μM cisplatin. After a 24 hr incubation, the cells were harvested in luciferase lysis buffer and the luciferase activity was assayed using Dual-Glo luciferase assay system with a GLOMAX™ 96 microplate luminometer (Promega).

### Curcumin-PLGA Nanoparticles (Nano-CUR)

PLGA nanoparticles (PLGA NPs) containing curcumin were prepared from curcumin and PLGA (50:50 lactide-glycolide ratio; inherent viscosity 1.32 dL/g in at 30°C) (Birmingham Polymers, Pelham, AL) using modified nano-precipitation technique [21]. In brief, 90 mg of PLGA was dissolved in 10 mL of acetone over a period of 3 hrs and 1 mg of curcumin was added to get a uniform PLGA-curcumin solution. This solution was drop wise added to 20 mL of aqueous solution containing 2% (wt./v.) poly(vinyl alcohol) (PVA) (M.W. 30,000-70,000) and 10 mg of poly-L-lysine (M.W. 30,000-70,000) (PLL), over a period of 10 min on a magnetic stir plate operated at 800 rpm. Within a few minutes precipitation can be observed in the aqueous layer. This suspension was stirred at room temperature for ~24 hrs to completely evaporate the acetone. Unentrapped curcumin was removed by centrifugation at 5,000 rpm on an Eppendorf Centrifuge 5810 R (Eppendorf AG, Hamburg, Germany) for 10 min. PLGA NPs with entrapped curcumin were recovered by ultracentrifugation at 30,000 rpm using Rotor 30.50 on an Avanti J-30I Centrifuge (Beckman Coulter, Fullerton, CA) and were subsequently lyophilized using a freeze dry system (-48°C, 133 × 10^-3^mBar Freeze zone^®^, Labconco, Kansas City, MO) and stored at 4°C until further use. Curcumin loading and release was estimated at 450 nm using Biomate 3 UV-vis spectrophotometer (Thermo Electron) as described earlier [22].

### Internalization of PLGA NPs

Cellular uptake of PLGA NPs was determined with nanoparticles prepared as described above but with 500 μg of fluorescein-5^'^-isothiocyanate (FITC) used in place of curcumin. The FITC loading in PLGA NPs was determined using UV-vis spectrophotometer [23] at 490 nm after extracting FITC for 1 day in acetone. FITC standards (1-10 μg/ml) were used for estimation of FITC in PLGA NPs. To determine the PLGA NPs uptake in A2780CP cells, 50,000 cells were plated in 4 well chamber slides and after 24 hrs the media was replaced with PLGA NPs (20 μg of FITC) diluted in media. After 6 hrs incubation with FITC-PLGA NPs, cells were washed twice in PBS, fixed with ice cold methanol for 10 min, washed with PBS and stained with DAPI (1:1000 dilution) (Invitrogen) to label the nucleus of the cells. Fluorescence microscope images were taken on an Olympus BX 51 microscope (Olympus, Center Valley, PA) equipped with an X-cite series (ExFo, Quebec Canada) excitation source and an Olympus DP71 camera.

### Anti-TAG-72 MAb conjugation to PLGA NPs

The feasibility of antibody conjugation was determined with PLGA NPs. The conjugation reaction was performed with anti-TAG-72 MAb (CC49) and PLGA NPs utilizing conjugation chemistry employing a reactive di-functional cross-linker, NANOCS NHS-PEG-NHS (MW 5,000) (NANOCS, New York, NY) at a ratio 1:20 (antibody to NPs), as shown in Figure 6F. Unconjugated anti-TAG-72 MAb was removed by ultra centrifugation. The antibody conjugation was confirmed by immunoblotting. The samples (5 μg of anti-TAG-72 MAb conjugated PLGA NPs, PLGA NPs or 2 μg free anti-TAG-72 MAb) were heated at 95°C for 5 min, cooled down to 4°C and centrifuged at 14,000 rpm for 3 min and supernatants were collected. Following gel electrophoresis and protein transfer, membranes were probed with a horseradish peroxidase- conjugated goat anti-mouse antibody and the signal was detected as described above.

### Statistical Methods

Analysis of variance (ANOVA) was followed by the student t-test with Bonferroni correction for multiple comparisons (to be considered significant, the p value must be less than 0.017 (0.05/3 = 0.017)). Normality of distribution, equal variance, ANOVA, and t-tests were performed using the statistical software package, JMP 8.0 (SAS, Carry, NC).

## Results

### Curcumin pre-treatment induces chemo/radio-sensitization in ovarian cancer cells

**Figure 1:**
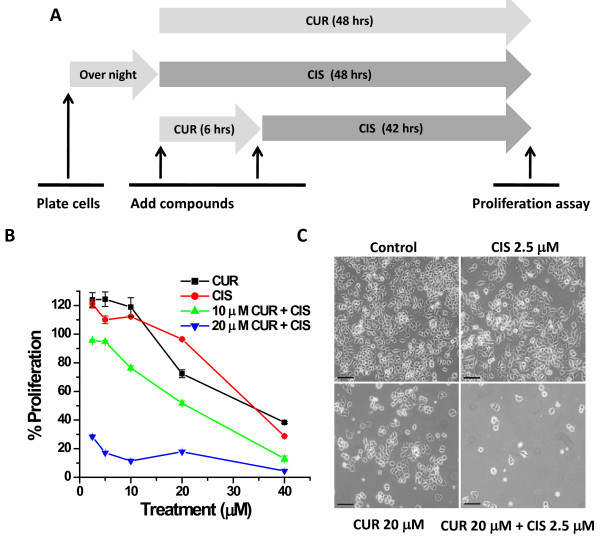
**Curcumin pre-treatment effectively lowers the cisplatin dose needed for inhibiting growth of cisplatin resistant A2780CP ovarian cancer cells**. **(A) Design of treatment method for curcumin sensitization followed by cisplatin treatment**. Cisplatin resistant ovarian cancer cells (A2780CP) were either treated with curcumin or cisplatin alone for 48 hrs, or pre-treated with curcumin for 6 hrs followed by cisplatin for an additional 42 hrs. **(B) Curcumin pre-treatment followed by cisplatin exposure decreases cell proliferation at lower doses of cisplatin**. A2780CP cells were treated with either 2.5-40 μM of curcumin (CUR) or cisplatin (CIS) alone for 48 hrs or pre-treated with 10 or 20 μM curcumin for 6 hrs followed by 2.5-40 μM of cisplatin treatment for 42 hrs (CUR + CIS). Cell proliferation was determined by MTS assay and normalized to control cells treated with appropriate amounts of vehicle (DMSO or DMSO-PBS). Data represent mean ± SE of 6 repeats for each treatment and the experiment was repeated three times. **(C) Phase contrast microscopic analysis reveals curcumin sensitization to cisplatin**. Phase contrast images of A2780CP cells treated with vehicle (DMSO, control), 2.5 μM CIS for 48 hrs, 20 μM CUR for 48 hrs, and 20 μM CUR for 6 hrs followed by 2.5 μM CIS for 42 hrs. Bar equals 100 microns.

To determine if curcumin could sensitize cisplatin-resistant ovarian cancer cells (A2780CP) to cisplatin treatment, we designed a curcumin pre-treatment strategy and compared individual treatments (curcumin or cisplatin) to a combination of treatments (curcumin and cisplatin) (Figure 1A). When used individually, curcumin and cisplatin have limited dose dependent anti-proliferative effects on A2780CP cells (Figure 1B, CUR + CIS). However, pre-treatment with 20 μM curcumin for 6 hrs followed by treatment with 2.5-40 μM cisplatin for an additional 42 hrs resulted in drastic cell growth inhibition compared to each agent alone (Figure 1B, CUR + CIS). The cisplatin sensitive ovarian cancer cell line, A2780 (the parental cell line of A2780CP), also showed increased sensitivity to cisplatin following pre-treatment with curcumin (data not shown). Additionally, a 6 hr pre-treatment with curcumin was more effective than treating the cells with curcumin and cisplatin simultaneously (data not shown). Of note, the MTS assay that is used to determine cell proliferation does not directly distinguish between induction of cell death or prevention of cell division; however, the result is clear that curcumin pretreatment dramatically increases the effects of cisplatin on ovarian cancer cells. Microscopic examination of treated cells revealed that 2.5 μM cisplatin did not change cell number or morphology and that 20 μM curcumin had a moderate decrease in cell number (Figure 1C). However, when pre-treated with 20 μM curcumin, 2.5 μM cisplatin drastically reduced the cell survival (Figure 1C).

**Figure 2:**
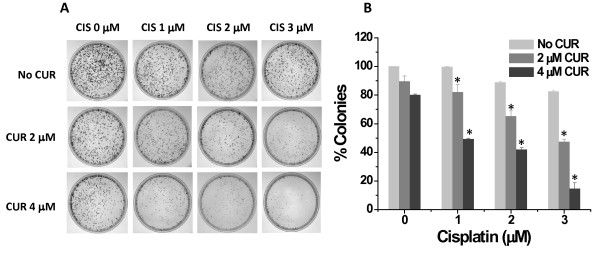
**Curcumin pre-treatment followed by cisplatin exposure reduces the clonogenic potential of A2780CP cells**. A2780CP cells were treated with the indicated amounts of curcumin or cisplatin alone or pre-treated with curcumin for 6 hrs followed by cisplatin and allowed to grow for 8 days. **(A) **Representative images of colony forming assays. **(B) **Colonies were counted and expressed as a percent of the DMSO vehicle control. Data represent mean of 3 repeats for each treatment (Mean ± SE; * p < 0.017, compared to the same cisplatin dose without curcumin).

**Figure 3:**
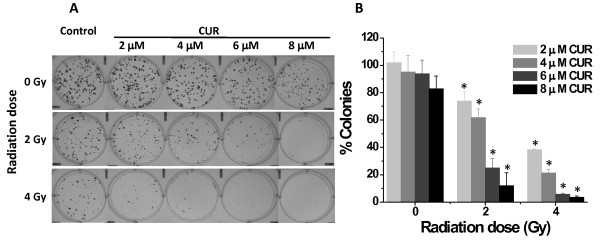
**Curcumin pre-treatment sensitizes cells to radiation exposure and reduces the clonogenic potential of A2780CP cells**. A2780CP cells were treated with the indicated amounts of curcumin or radiation alone or pre-treated with curcumin for 6 hrs followed by radiation exposure and allowed to grow for 8 days. **(A) **Representative images of colony forming assays. **(B) **Colonies were counted and expressed as a percent of each respective dose of radiation. Data represent mean of 3 repeats for each treatment (Mean ± SE; * p < 0.017, compared to the same dose of curcumin with no radiation exposure).

To determine the long-term effect of chemo-sensitization with curcumin pre-treatment, we performed colony forming assays with cells either treated individually with curcumin or cisplatin, or with a 6 hr pre-treatment of curcumin followed by cisplatin treatment (Figure 2). Pre-treatment of cells with curcumin (2 and 4 μM) followed by cisplatin (1-3 μM) resulted in a greater inhibition of colony formation than each agent alone (Figure 2). Due to the prolonged incubation after drug treatment(s), it is not surprising that lower doses of cisplatin/curcumin had significant effects compared to the 48 hr proliferation assay. Further, we have determined the effect of curcumin pre-treatment on ovarian cancer cell's sensitivity to radiation. Pre-treatment of cells with curcumin (2-8 μM) followed by radiation exposure (2-8 Gy) resulted in greater inhibition of colony formation than curcumin or radiation alone (Figure 3). From these data, it is apparent that curcumin can induce chemo/radio-sensitization in ovarian cancer cells and may considerably lower the minimum effective dose of cisplatin or radiation treatment.

### Curcumin pre-treatment modulates the expression of pro-survival/pro-apoptosis proteins

**Figure 4:**
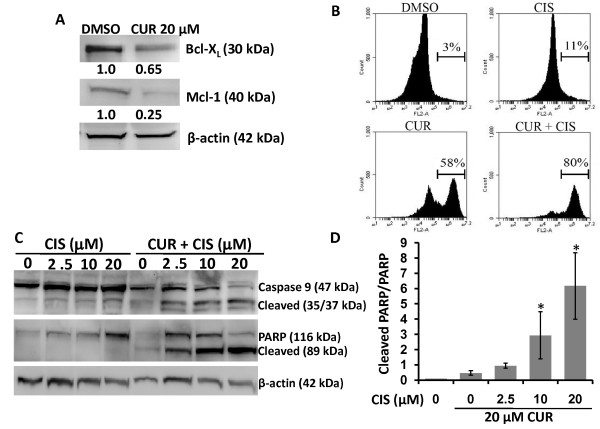
**Curcumin treatment alters the expression of pro-survival and pro-apoptosis related proteins**. **(A) Curcumin decreases the expression of Bcl2 family of pro-survival proteins during 6 hr pre-treatment**. A2780CP cells were treated with 20 μM curcumin for 6 hrs and protein lysates were collected and analyzed by immunoblotting for Bcl-xl, Mcl-1 and β-actin. Appropriate bands were quantified by densitometry, normalized to β-actin, scaled to the DMSO control and expressed as relative expression levels (number beneath the blots). **(B) Curcumin pre-treatment followed by low dose cisplatin increases percent of Annexin V positive cells**. A2780CP cells treated as indicated with vehicle (DMSO), curcumin (20 μM) or cisplatin (5 μM) only or pre-treated with curcumin (20 μM) followed by cisplatin (5 μM) treatment. After 48 hrs, adherent and attached cells were stained with Annexin V-PE and analyzed by Flow Cytometry. Representative histograms are shown for 1 of 3 similar experiments. **(C and D) Curcumin pre-treatment promotes the induction of apoptosis by cisplatin**. A2780CP cells were treated as indicated for a total of 48 hrs and protein lysates were collected and analyzed by immunoblotting for caspase 9 and PARP. **(D) **Bands for full length and cleaved PARP were quantified by densitometry, normalized to β-actin and calculated as a ratio of cleaved PARP to full length PARP. Data represent mean of 3 repeats for each treatment (Mean ± SE, * p < 0.017, compared to curcumin only).

To examine the possible molecular mechanisms by which curcumin induces chemo/radio-sensitization effects in A2780CP cells, we examined the expression pattern of pro-survival Bcl-2 family members expressed by A2780CP cells (data shown for Bcl-X_L _and Mcl-1). Following a 6 hr pre-treatment with 20 μM curcumin, the expression of Bcl-X_L _and Mcl-1 was decreased (Figure 4A), which would suggest increased sensitivity to apoptosis. Hence, we sought to determine if cell death was occurring through an apoptotic pathway. Following curcumin pre-treatment, both adherent and floating cells were collected, stained with Annexin V-PE and analyzed by flow cytometry. Curcumin pre-treatment followed by cisplatin treatment resulted in a substantial increase in Annexin V positive cells (Figure 4B), indicating induction of cell death *via *an apoptotic pathway. We confirmed this observation by probing for the expression of PARP and caspases 3, 7 and 9, as proteolytic cleavage and subsequent activation of these molecules activate apoptotic pathways. A2780CP cells pre-treated with curcumin and then treated with cisplatin showed higher levels of cleaved caspase 9, in contrast to cells treated with curcumin or cisplatin alone (Figure 4C). Additionally, the expression level of full-length caspase 3 and 7 was decreased, suggesting cleavage and activation of the caspase pathway; however, cleaved products of caspase 3 or 7 were not detectable (data not shown). Furthermore, we also assessed treated cells for cleavage of PARP, a classic marker for apoptotic cells. Pre-treatment with curcumin followed by cisplatin exposure resulted in increased PARP cleavage in a dose dependent manner, while cisplatin alone was unable to induce PARP cleavage even at the highest dose (Figure 4C and 4D). We detected an increase in full length PARP after 20 μM cisplatin treatment (Figure 4C), which could be an indication of the cancer cell's attempt to survive cisplatin induced DNA damage by increasing DNA repair proteins, such as PARP. However, in curcumin pre-treated cells, cisplatin exposure resulted in a significant (p < 0.05) increase in PARP cleavage, indicating the induction of apoptosis.

### Curcumin suppresses β-catenin activity

**Figure 5:**
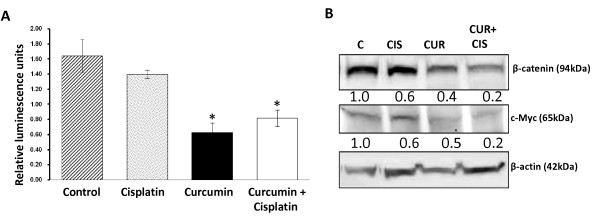
**Curcumin inhibits nuclear β-catenin signaling**. **(A)Curcumin inhibits β-catenin transcription activity**. A2780CP cells were transiently transfected with TOPFlash or FOPFlash and co-transfected with Renilla luciferase to determine β-catenin/TCF transcription activity. The cells were treated with 20 μM curcumin, 5 μM cisplatin or a 6 hr pre-treatment with 20 μM curcumin followed by treatment with 5 μM cisplatin. After 24 hrs of incubation, cell lysates were collected and probed for luciferase activity. Treatment of A2780CP cell line with 20 μM curcumin resulted in over a 60% reduction in β-catenin activity (Mean ± SE, n = 3, *p value p < 0.017, compared to control). **(B) Curcumin treatment reduces overall β-catenin and c-Myc protein levels**. A2780CP cell lines were treated as in (A), protein lysates were collected and analyzed by immunoblotting for β-catenin, c-Myc and β-actin. Protein bands were quantified by densitometry, normalized to β-actin, scaled to the DMSO control and expressed as relative expression levels (number beneath the blots). Curcumin treatment caused a 50% reduction in β-catenin and c-Myc levels.

Inappropriate activation of β-catenin is linked with the development of a wide variety of cancers, including melanoma, colorectal and prostate cancer [24,25]. Additionally, deregulation of the Wnt/β-catenin pathway has also been shown in ovarian cancer [26,27]. As a modulator of the Wnt signaling pathway, β-catenin functions as a transcription factor that is translocated into the nucleus where it binds with the TCF transcription factor and up-regulates the expression of cell survival genes such as c-Myc and c-Jun, which as a result, enhances cell proliferation in cancer cells. It has also been shown that β-catenin activity can also inhibit apoptosis in cancer cells [28-31]. Therefore, we sought to investigate the effects of curcumin treatment on nuclear β-catenin function in cisplatin resistant ovarian cancer cells using TOPFlash reporter assay. The cells were treated with either curcumin, cisplatin or a 6 hr pre-treatment with curcumin followed by treatment with cisplatin. After 24 hrs of incubation, cell lysates were collected and analyzed for β-catenin transcription activity. While treatment of the cells with cisplatin caused no change in the β-catenin activity, curcumin treatment repressed the β-catenin mediated transcription activity by 60% (Figure 5A). The combination of curcumin and cisplatin also reduced β-catenin activity to similar levels as when treated with curcumin (there is not a significant difference between curcumin only and combination treatment with curcumin and cisplatin). To further investigate curcumin mediated repression of β-catenin activity, we analyzed the overall expression of β-catenin levels and the expression of a downstream target of nuclear β-catenin signaling (c-Myc) by Western blotting. Curcumin treatment leads to ~50% reduction in β-catenin and c-Myc protein levels (Figure 5B). This data suggest that curcumin treatment attenuates nuclear β-catenin signaling, which is known to play a significant role in cancer cell proliferation.

### PLGA nanoparticle formulation of curcumin (Nano-CUR) effectively inhibits ovarian cancer cells growth

**Figure 6:**
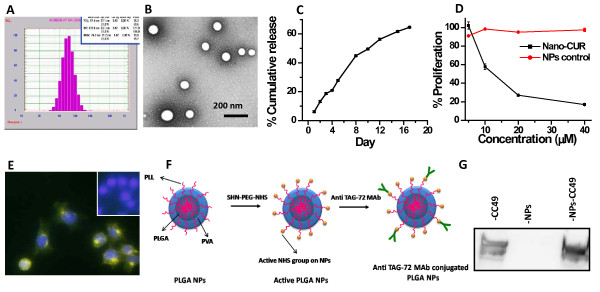
**Characterization of PLGA-nanoparticle (NP) containing curcumin (Nano-CUR) and its *in vitro *therapeutic efficacy**. **(A and B) Nano-CUR particles are an appropriate size of ~70 nm**. Nano-CUR size was determined by **(A) **dynamic light scattering (DLS) and **(B) **transmission electron microscopy (TEM). **(C) Nano-CUR formulation demonstrates sustained release of curcumin**. Cumulative release of curcumin from PLGA NPs was determined by UV spectrophotometer at 450 nm over a period of 18 days. **(D) Nano-CUR effectively inhibits the growth of cisplatin resistant ovarian cancer cells**. A2780CP cells were treated with Nano-CUR (5-80 μM) or PLGA NPs without curcumin (NPs control) for 48 hrs. Cell proliferation was determined by MTS assay and normalized to control cells treated with vehicle (PBS). **(E) A2780CP cells internalize PLGA-NPs**. A2780CP cells were incubated with FITC-PLGA NPs for 6 hrs and analyzed by fluorescent microscopy. Original magnifications 400×. Inset image represents PLGA NPs no FITC. **(F) Strategy used for antibody conjugation of PLGA-NP for targeted delivery of curcumin to ovarian cancer cells. (G) PLGA-NPs can be conjugated with anti-TAG-72 MAb (CC49)**. PLGA-NPs were incubated with anti-TAG-72 MAb. Nano-immunoconjugates were run on 10% SDS-PAGE, transferred to the PVDF membrane and were probed with an anti-mouse secondary antibody as indicated.

While we have shown that curcumin has effective chemo/radio sensitization effects in ovarian cancer cells, low water solubility and poor pharmac okinetics greatly hamper curcumin's *in vivo *therapeutic efficacy. Therefore, we decided to synthesize a PLGA nanoparticle (NP) formulation of curcumin, which is expected to improve bioavailability *in vivo *[32,33]. Following synthesis, Nano-CUR was physically characterized by both dynamic light scattering (DLS) and transmission electron microscopy (TEM). The average size of Nano-CUR was observed to be ~72 nm by DLS (Figure 6A) and 70 ± 3.9 nm by TEM (Figure 6B). Additionally, curcumin is released from PLGA NPs in a controlled fashion, which may be useful for sustained and long term delivery of curcumin for ovarian cancer treatment (Figure 6C). Following particle characterization, we examined the *in vitro *therapeutic efficacy of Nano-CUR and found that Nano-CUR treatment effectively inhibited proliferation of ovarian cancer cells (Figure 6D). Additionally, PLGA NPs are efficiently internalized by A2780CP cells (Figure 6E). Further, to verify that these nanoparticles are capable of antibody conjugation for targeted delivery specifically to ovarian cancer cells, we conjugated nanoparticles with anti-TAG-72 monoclonal antibody (MAb) (Figure 6F). TAG-72, a tumor-associated glycoprotein, is over-expressed in various tumors, including ovarian cancer [34]. Western blot analysis of conjugated PLGA NPs revealed that anti-TAG-72 MAb was effectively conjugated to PLGA NPs (Figure 6G). These data suggest that, in the future, targeted delivery of curcumin specifically to tumors will be possible. This strategy will improve the therapeutic efficacy of curcumin and will be useful for specific chemo/radio-sensitization of cancer cells.

## Discussion

Most ovarian cancers initially respond well to current treatment modalities, but the majority of patients will experience recurrence. Unfortunately, almost all recurrent ovarian cancers eventually develop resistance to platinum based treatment. Tumors with intrinsic or acquired resistance may have various altered characteristics, including: (a) altered membrane transport properties, (b) altered expression of target enzymes, (c) promotion of DNA repair, (d) degradation of drug molecules, and (e) generalized resistance to apoptosis [35-37]. A promising strategy for improving current ovarian cancer therapy is to employ a chemo/radio-sensitizer along with chemo/radiation therapies.

Curcumin is an excellent candidate as a chemo/radio sensitizer and has been shown to have *in vitro *chemo-sensitization effects for cervical cancer and radio-sensitizing effects for prostate cancer [38,39]. However, curcumin's utility for ovarian cancer treatment has not been fully explored [40-42]. Chirnomas et al. reported that a functional Fanconi anemia (FA)/BRCA pathway limits sensitivity to cisplatin and that curcumin can inhibit this pathway, leading to increased sensitivity to cisplatin treatment in ovarian cancer cells [41]. Our study shows that a 6 hr pre-treatment with curcumin effectively sensitized cisplatin resistant ovarian cancer cells to the cytotoxic effects of cisplatin, at doses at least 10 times lower compared to cisplatin treatment alone. Using clonogenic assays, we assessed the long term effects of curcumin pre-treatment along with cisplatin treatment or radiation exposure. We found that curcumin pre-treatment followed by cisplatin or radiation exposure dramatically reduced colony formation compared to either treatment alone. Curcumin pre-treatment clearly lowers the dose of cisplatin and radiation treatment needed to suppress the growth of ovarian cancer cells.

Apoptosis is normally a carefully balanced system of checks and balances. In cancer cells, often the balance has been tilted to be more resistant to the initiation of apoptosis. Over-expression of pro-survival Bcl2 family members is common in many types of cancer and has been correlated with decreased sensitivity to chemotherapy and radiation [43]. We found that curcumin pre-treatment reduced the expression of two pro-survival proteins, Bcl-X_L _and Mcl-1, potentially allowing curcumin treated cells to undergo apoptosis upon cisplatin treatment. Indeed, pre-treatment with curcumin followed by cisplatin increased the percent of Annexin V positive cells and increased the amount of cleaved caspase 9 and PARP, as compared to cisplatin or curcumin alone, indicating that curcumin pre-treatment followed by cisplatin enhanced apoptosis.

Curcumin treatment reduced the transcriptional activity and expression level of β-catenin. The β-catenin pathway is known to be disrupted in a variety of cancers, including ovarian cancer. Activation of the β-catenin signaling pathway leads to nuclear localization of β-catenin which interacts with the TCF transcription factor and modulates the expression of a wide range of proto-oncogenes. The functions of these responsive genes are thought to increase proliferation and recent studies have also suggested that β-catenin signaling may also inhibit apoptosis [28-31]. Taken together, these results suggest that curcumin pre-treatment increases the effectiveness of cisplatin treatment in cisplatin resistant cells by increasing the sensitivity of cells to apoptotic pathways and modulating nuclear β-catenin signaling.

Curcumin is in early phase clinical trials for various types of cancers [44]. Curcumin is remarkably well tolerated and has no toxicity issues [45,46], but it has limited bioavailability and poor pharamacokinetics [47,48]. To improve curcumin's *in vivo *effectiveness we have developed a PLGA nanoformulation of curcumin. Nanoparticles can deliver anti-cancer drugs to the site of disease with an antibody targeting approach; however, major drawbacks include interaction with serum proteins (causing opsonization), clearance by the reticuloendothelial system, and non specific accumulation in organs [49]. To counter these difficulties and to extend the circulation time of nanoparticles in the blood, nanoparticles may be modified with inert hydrophilic polymers, such as poly(ethylene glycol) and poly(vinyl alcohol). In addition, formulating a small particle size (less than 100 nm) with high antibody conjugation efficiency will further enhance the ability to target tumors efficiently [50]. In our current study, we have developed PLGA nanoparticles which are made using FDA approved polymer (PLGA) and coated with poly(vinyl alcohol). The formulated Nano-CUR effectively inhibits proliferation in cisplatin resistant ovarian cancer cell lines. The size of these PLGA NPs were formulated to ~70 nm which is an important parameter for enhancing the circulation life time and ensuring diffusion of particles into tumor sites. Recent literature suggests that antibody conjugated nanoparticles could efficiently deliver chemotherapeutic drugs to the tumor site [51-53]. Accordingly, we have shown efficient conjugation of anti-TAG-72 MAb to PLGA NPs with our conjugation chemistry for targeting applications. Targeted delivery of curcumin will improve the therapeutic efficacy of curcumin and will be useful for specific chemo/radio-sensitization of cancer cells. Overall, the results of this study suggest that curcumin pre-treatment induces chemo/radio-sensitization in ovarian cancer cells *via *modulating pro-survival cellular signaling and nanoparticle mediated curcumin delivery may further improve the therapeutic efficacy of curcumin.

## Conclusion

We report that curcumin acts as a chemo/radio-sensitizer by modulating the expression of pro-survival proteins and increasing apoptosis in response to a low dose of cisplatin. Nanoparticle mediated curcumin delivery will further improve the sensitization and therapeutic capabilities of curcumin. This study demonstrates a novel curcumin pre-treatment strategy that could be implemented in pre-clinical animal models and in future clinical trials for the effective treatment of chemo/radio-resistant ovarian cancers.

## Competing interests

The authors declare that they have no competing interests.

## Authors' contributions

MMY designed and performed MTS assays, colony formation assays, Western blotting, and synthesis of PLGA NP formulations. DM participated in the design of the study, provided technical support and performed flow cytometry analysis. DM and MMY drafted the manuscript together. VS performed and analyzed the β-catenin assays and participated in manuscript preparation. SCC and MJ participated in the inception of the idea, experimental design, and revision of the manuscript. All authors read and approved the manuscript.
